# WHO Southeast Asia regional flagships – building beyond global frameworks

**DOI:** 10.1016/j.lansea.2023.100314

**Published:** 2023-10-29

**Authors:** Swarup Sarkar

**Affiliations:** University of Gothenburg, Sweden

The General Programme for Work of WHO, during its 12th iteration, included a results-based framework for the first time.^1^ At the same time, the WHO SEAR Regional Director, moving from a process-based framework, introduced an outcome-based prioritisation in 7 areas, later extending to 8 flagship areas.^2^ This commentary examines the positive outcomes and challenges faced by the flagships.

## Regional adaptation led to ownership

The flagship programs create prioritisation, a sense of urgency, tangible goals, and greater scope for alignment with national and regional priorities, making it appealing to local political leaders and incentivising further investment. This is evident through 21 elimination programmes validated during this time that no other region could achieve. In most cases, these elimination programmes were seen as a national achievement by the heads of state.^1^ So were the emergency responses to physical disasters. However, a few flagships did not lead to success. Here are a few examples of successes and failures.

## Flagship programs need dynamic policy adjustment, sub-national analysis; and equity in consideration

When the regional flagship was adopted initially, TB was not included despite being the largest killer infectious disease in the region. Subsequently, an analysis by SEARO in 2015 concluded that none of the countries would attain SDG targets of TB by 2030 unless new strategies were adopted, which led to the addition of TB as a flagship and influenced the global strategy.^3^ One of the criteria for choosing a flagship disease is to assess its technical feasibility, which varies between diseases and countries.

On the other hand, the declaration of achievements on leprosy at the national and regional levels did not consider huge sub-national areas of high prevalence. This led to a plateauing of case detection with at least 100,000 cases, stagnation of resources, and a continued disproportionate burden of leprosy on tribal populations.^4^^,^^5^

Elimination goals of malaria in Bhutan, with just a handful of cases, got repeatedly postponed. Filariasis and Kala Azar's goals were also shifted many times in several countries. Translation of priorities into local actions, quality products of international standard for localised diseases, specifically those with catastrophic consequences, require investment in the sub-national presence of WHO, real-time use of digital technology, civil society presence, and south-based think tanks and collaborating centres.^6^^,^^7^

The Thailand-based Asia Epidemic Modelling Centre has not only produced Asia-specific policy for high-risk group prioritisation but also shaped the global response by prioritising a relatively small proportion of the population like sex workers, people who inject drug and men who have sex with men and their partners. Unfortunately, most policy centres are based in the Global North, and despite the game-changing work of SEARO on TB through modelling, the region has yet to set up such a centre.^7^ All the diagnostic tools still in use for kala-azar have not yet been validated, and adoption and elimination remain challenging. In contrast, TB genetic tools have been quickly validated and pre-qualified, and POC technology has been made widely available, although scaling up is yet to be achieved.

## Costing and resource gap analysis on a real-time basis

Despite being the first of these flagship programs, AMR also had a ministerial declaration and commitment from SEARO countries that did not see significant resources allocated without a fully costed programme. Leprosy and most NTD programmes have not been costed. Indeed, the resources of HIV swelled after the first resource need study came before the UN General Assembly special session on AIDS backed up by advocacy by the affected people.^8^ Similarly, the success of resource mobilisation for TB (from 500 million to 1.2 billion USD) came from combining the highest level of advocacy with resource gap analysis.

## Better use of country cooperation strategy

Country co-operation strategy (CCS), a tool to guide resource allocation of WHO, is often seen by the countries as a means to mobilise additional resources. As a result, diseases that have resources available from other sources are neglected, even if they are insufficient. Resources available from GFATM lead to a lack of prioritisation of AIDS, TB and Malaria by most countries in the Country Cooperation Framework despite the resource gaps. Malaria is the only disease among the SDG 3.3 targets that is likely to be achieved in the SEA Region on time, but has never been explicitly captured in the regional flagship and most CCS.^9^

Therefore, flagships, though proven to have vast potential for success, require additional ground-level work. These are not necessarily biomedical interventions but essential enablers that could be captured in future iterations, a few of which are^6^:

o Framework for multisectoral coordination at national and subnational level
o Involvement of civil society groups in the design and implementation of the programme
o Addressing costs and cultural barriers to access to services
o Fast route tracking of market entry of point-of-care products and other tools
o Operationalisation of real-time digital surveillance of disease and the response
o Investment of south-based think tank, policy work linked to flagships

Some of these programmes currently missed in the WHO GPW framework have earlier been identified as social determinants and essential enabling interventions programme in the UNAIDS programme and newer organisations like UNITAID have been established.^10^^,^^11^

Future flagship programmes should capture these, and a separate stream of work may be developed on the above areas. The current Series has been shy of WHO's weaknesses and framework processes. A robust institutional culture of transformation and self-criticism are essential for the WHO to meet the needs of the Member States and the health challenges of people left behind.

To summarise, the success of the flagship programs is contingent upon various factors such as priority setting based on disease burden analysis, robustness of technical strategies, feasibility of achievement of targets, estimation of resource need, adequate resource allocation based on resource gap analysis, availability of point-of-care technology, and people-centred service delivery mechanisms. Additionally, enabling interventions that strengthen civil society, investment in quality product development process, pre-qualification, and regulatory mechanism capacity also play a crucial role. The forthcoming 14th Global Programme of Work (GPW) of the World Health Organization (WHO) can benefit from these lessons learnt.

## Contributors

Swarup Sarkar has conceptualised and drafted the manuscript.

## Declaration of interests

Author has been associated with some of these flagships of the WHO SEARO as a staff member and as a consultant.
